# The Minha Casa Minha Vida social housing programme and leprosy in Brazil: an analysis of the 100 Million Brazilian Cohort (2010–2015)

**DOI:** 10.1186/s12889-025-22701-8

**Published:** 2025-08-04

**Authors:** Camila S. S. Teixeira, Júlia M. Pescarini, Mauro N. Sanchez, Andrêa Jacqueline F. Ferreira, Rosemeire L. Fiaccone, Maria Yury Ichihara, Renzo Flores-Ortiz, Elizabeth B. Brickley, Peter Craig, Alastair H. Leyland, Srinivasa Vittal Katikireddi, Maria Lucia F. Penna, Gerson O. Penna, Rita de Cássia Ribeiro-Silva, Maurício L. Barreto

**Affiliations:** 1 Oswaldo Cruz Foundation - Gonçalo Moniz Institute, Center for Data and Knowledge Integration for Health (CIDACS), Salvador, Bahia 41 745 - 715 Brazil; 2 Institute of Public Health, Federal University of Bahia (UFBA), Salvador, Brazil; 3 Faculty of Epidemiology and Population Health, London School of Hygiene & Tropical Medicine, London, United Kingdom; 4 Tropical Medicine Center, University of Brasilia (UnB), Brasília, Brazil; 5 Institute of Mathematics and Statistics, Federal University of Bahia (UFBA), Salvador, Brazil; 6 Institute of Health & Wellbeing, University of Glasgow, Glasgow, Scotland; 7 Department of Epidemiology and Biostatistics, Fluminense Federal University (UFF), Rio de Janeiro, Brazil; 8 Fiocruz Government School, Oswaldo Cruz Foundation Brasilia, Brasília, Brazil; 9 School of Nutrition, Federal University of Bahia (UFBA), Salvador, Brazil

**Keywords:** Communicable diseases, Leprosy, Inequality, Socioeconomic factors, Social housing

## Abstract

**Background:**

Ensuring housing interventions can contribute to improved living conditions which are strong socioeconomic determinants of leprosy. We estimated the association between the social housing programme Minha Casa Minha Vida (MCMVP) and leprosy new cases.

**Methods:**

We followed families registered in the 100 Million Brazilian Cohort linked with MCMVP receipt and nationwide registries of leprosy between 2010 and 2015. We used Cox regression weighted by stabilized inverse probability of treatment weighting (IPTW) to assess the hazard ratio (HR) for the effect of MCMVP on leprosy. Weights were obtained by propensity score using demographic and socioeconomic covariates at baseline. Sensitivity analyses were done considering potential delays to receiving MCMVP, municipality of residence population size and by controlling by the baseline risk of leprosy among potential recipients.

**Results:**

We followed up 24,584,768 individuals, of which 618,883 (2.5%) were MCMVP recipients, and detected 8,874 new leprosy cases during the study period. Leprosy incidence was higher among MCMVP recipients (13.32/100,000 pyr; 95%CI = 11.45–15.49) compared to non-recipients (11.72/100,000 pyr; 95%CI = 11.47–11.97). MCMVP recipients had higher leprosy incidence (HR = 1.66; 95%CI = 1.34–2.06), compared to non-recipients. Point estimates were lower when considering a delay of 6 or 12 months to moving into the new household (HR = 1.53; 95%CI = 1.20–1.95 and HR = 1.37; 95%CI = 1.05–1.78, respectively), in small/medium municipalities (≤ 300,000 inhabitants) (HR = 1.95; 95%CI = 1.51–2.52), and higher among individuals who subsequently became MCMVP beneficiaries before receiving the benefit (HR = 2.29; 95%CI = 1.93–2.72).

**Conclusions:**

This study found a higher risk of leprosy associated with MCMVP that may reflect reverse causality. Our findings suggest the programme is, in fact, reaching the most vulnerable individuals, as intended in its objectives. Besides, the higher risk of leprosy among MCMVP beneficiaries even before receiving the benefit observed in sensitivity analysis may reflect residual confounding factors related.

## Background

Healthy household is a reflect of good living conditions within the home and the neighbourhood as well as housing affordability [1, 2]. Shortfalls in the quality and availability of housing present a significant public health challenge [2] and may contribute to the persistent burden of infectious diseases of poverty [3].

Leprosy is an infectious disease of poverty, caused by *Mycobacterium leprae* (*M. leprae*), that can lead to severe disabilities in the hands, feet and eyes if left untreated [4]. More than 200,000 cases of leprosy are diagnosed per year globally, concentrated in areas of greater social vulnerability in middle- and low-income countries [5]. Brazil has the second highest number of leprosy cases worldwide, with a new case detection rate (NCDR) of 11.21/100,000 inhabitants in 2023 [6]. Pockets of high leprosy burden likely arise due to a combination of increased exposure to infectious cases and issues related to surveillance and control actions for early detection and treatment [7]. In addition, poverty and deprivation at the household and neighbourhood levels, such as precarious housing infrastructure, crowding, and lack of access to basic sanitation and social services are factors known to be extensively associated with leprosy risks [5, 8, 9].

Social housing programmes offer a direct strategy for reducing social inequalities and improving health and has shown to reduce the occurrence of non-communicable diseases (e.g., cardiovascular diseases and mental illness) [10], but there is little available evidence regarding their impacts on infectious diseases [10, 11]. In Brazil, the ‘Minha Casa Minha Vida’ programme (MCMVP) was implemented in 2009 by the Brazilian Government to provides access to home ownership through different sub-programmes and is considered to be the largest social housing programme in Latin America [12].

For this study, we hypothesized that the MCMVP could contribute to reducing leprosy new cases, since the improvement of housing conditions has the potential to mitigate respiratory transmission of the infectious agent [5, 8, 9]. Using data from national health and administrative databases from Brazil, this study aimed to estimate the effect of MCMVP on leprosy new case detection.

## Methods

### Study design and data sources

This cohort study used baseline data on individuals in The 100 Million Brazilian Cohort linked with leprosy records from the Notifiable Diseases Information System (*Sistema de Informação de Agravos de Notificação*, SINAN-leprosy) and housing records from the MCMVP.

The 100 Million Brazilian Cohort [13] was built by the Center for Data and Knowledge Integration for Health (CIDACS), Oswaldo Cruz Foundation, Salvador, Brazil, using administrative records from the *Cadastro Único para Programas Sociais* (CadÚnico), a national registry for social assistance programmes for low-income families (https://cidacs.bahia.fiocruz.br/en/). The baseline includes geographic and socioeconomic information of individuals followed between 1 st January 2001 and 31 st December 2015.

Newly detected cases of leprosy within the baseline were identified through the linkage with SINAN-leprosy (2007–2015). SINAN records include sociodemographic and clinical features of patients at the time of diagnosis (date of diagnosis; WHO classification, based on the number of skin and nerves injuries (i.e., paucibacillary (PB) or multibacillary (MB)); grade of disability at diagnosis, estimated by sensory and motor functions of the eyes, hands and feet (i.e., Grade 0, 1, or 2).

MCMVP database (2010–2017) contains sociodemographic characteristics of beneficiaries (i.e., date of birth and family income), contract signing dates for housing receipt and municipality. MCMVP was implemented based on monthly familial income in three categories up to 2015: range 1 (up to BRL 1,800.00; up to 3 × minimum wage), range 2 (up to BRL 4,000.00) and range 3 (up to BRL 9,000.00) [12]. Our study focus exclusively in range 1 which families are benefited through a lottery carried out by municipality [12], but data of lotteries are not available at national level. Within range 1, we also focus in housing funded by the Residential Lease Fund (*Fundo de Arrendamento Residencial* – FAR) (MCMVP-FAR) for urban areas [12].

#### Linkage

The 100 Million Brazilian Cohort was linked with MCMVP through deterministic data linkage using the social identification number (*Número de Identificação Social* – NIS). SINAN-leprosy registries were deterministically linked to the cohort using the CIDACS-RL linkage tool [14], based on five individual identifiers: *name*, *date of birth*, *sex*, *mother's name* and *municipality of residence*. Linkage accuracy [15] shown the best performance score limit selected for use in full linkage (≥ 0.92) reached a specificity of 0.89 (95% CI 0.88–0.90) and sensitivity of 0.91 (95% CI 0.90–0.92).

### Study participants

**Fig. 1:**
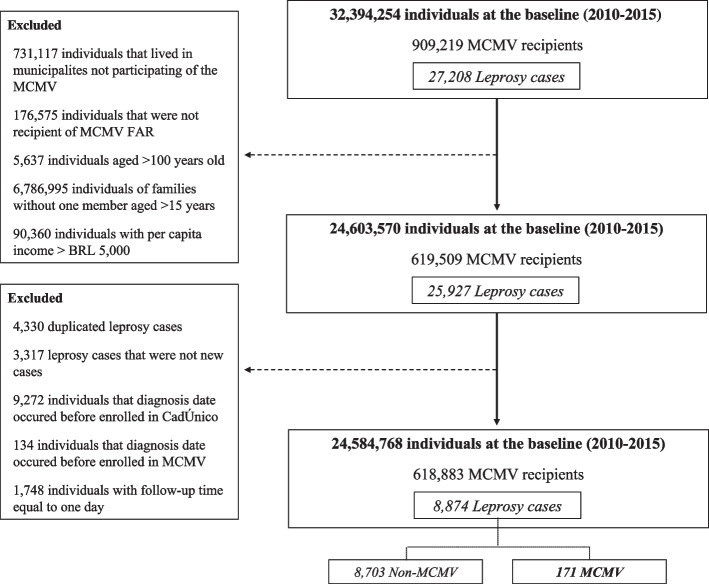
Selection of the study population from The 100 Million Brazilian Cohort, 2010–2015

The study population includes individuals who entered the 100 Million Brazilian Cohort between 2010 and 2015, considering the first application to the CadÚnico, that lived in municipalities participating of the MCMV and that received only MCMVP-FAR. We excluded (i) individuals > 100 years old, (ii) without a household member aged > 15 years, (iii) registered as a leprosy case prior to entering the cohort or before receiving MCMVP benefit, (iv) leprosy patients that had repeated entries in SINAN, considering the last diagnosis date for this exclusion; and (v) with follow-up time equal to one day (Fig. 1).

Study participants were followed up from the date of the entry in the cohort until the detection of a new leprosy case or until 31 st December 2015.

### Variables

We defined the detection of new leprosy cases as the main outcome. Individuals who received the MCMVP housing before the diagnosis of leprosy were considered exposed, while individuals who did not receive MCMVP were considered unexposed. We considered the date of contract signing as a proxy for the start of the exposure time. Other covariates included baseline sociodemographic characteristics (i.e., sex, age, self-reported race/ethnicity, education and participation in the conditional cash transfer ‘*Bolsa Família*’ programme – BFP), household conditions (i.e., wall construction materials, water and electricity supplies, and waste disposal), and geographic Brazilian region.

### Data analysis

We compared recipients and non-recipients of MCMVP using standardized differences, considering values of differences > 0.20 to represent a meaningful difference [16].

Leprosy incidence was estimated as the leprosy new case detection rate (NCDR) per 100,000 person-years at risk (pyr) by MCMVP benefit status and leprosy clinical features.

We used Cox proportional hazards model weighted by the inverse probability of receiving the treatment (IPTW) [17] to estimate the hazard ratios (HR) and 95% CI for the effect of the MCMVP on leprosy detection. MCMVP benefit was included as a time-dependent variable. To calculate the weights, we first estimated the propensity scores (*ps*) of receiving MCMVP benefits using a fully adjusted logistic regression model including all of the sociodemographic, household and geographic covariates as well as the year of study entry using a complete case approach (i.e., restricted to individuals without missing data for any of the covariates). Thereafter, we weighted non-recipients with the baseline probability of selecting a treatment (i.e., a model with only the MCMVP exposure) divided by one minus the *ps*. Estimation for recipients considered the marginal probability of receiving the treatment divided by the *ps*, which is the change in the probability that the outcome occurs as the risk factor changes by 1 unit considering all the explanatory variables of the *ps*.

### Sensitivity analysis

We performed two sensitivity analysis to test the robustness of our findings given different assumptions. To assess the leprosy incidences among individuals who subsequently became MCMV recipients, we used data from the period before the MCMV program (2006–2009) to estimate the NCDR per 100,000 pyr by MCMVP benefit status and leprosy clinical features as in main analysis. Further, to account for the possibility that some individuals might have moved into MCMVP housing a long timer after the contract signing date, we also performed the analysis considering the begin of exposure time for at the 6 th month and at the 12 th months after of the signature of the contract. To test if the effect of MCMVP varied due different patterns of coverage and distribution of health services around it, we stratified the analysis by the population size of the individual municipality of residence (i.e., ≤ 300,000 and > 300,000 inhabitants). Additionally, to test if beneficiaries had a higher baseline risk of developing leprosy due to uncontrolled confounding, we estimated the association between the MCMVP and leprosy detection among those individuals who subsequently became MCMVP beneficiaries before receiving the MCMVP benefit. For this analysis, follow-up for MCMVP beneficiaries begun in 2006 and ended when they received the programme.

All analyses were performed using Stata version 15.1 (*Stata Corporation,* College Station, Texas, USA).

### Ethics

This study was done according to the Declaration of Helsinki and Brazilian research regulations and was approved by the ethics committee from Gonçalo Muniz Institute – Oswaldo Cruz Foundation Bahia, under the project protocol number 1.612.302, and University of Glasgow, Medical, Veterinary and Life Sciences College (200190001). The cohort profile of the 100 Million Brazilians Cohort is available in the publications referenced in the article and further material is available at: https://cidacs.bahia.fiocruz.br/en/platform/cohort-of-100-million-brazilians. Data were stored on secure servers on the Cidacs Big Data Integrated Platform as described at: https://cidacs.bahia.fiocruz.br/o-cidacs-e-a-lgpd/. No personally identifiable information was kept in the dataset used for analysis.

## Results

Between 2010 and 2015, this study followed up 24,584,768 individuals, of which 618,883 (2.5%) received MCMVP during the follow up, accounting for 1,268,397.7 person-year at risk (pyr) (median = 1.9 years, IQR = 0.8–3.3). During follow-up, 8,874 individuals were diagnosed as new leprosy cases, of whom 171 (1.9%) were MCMVP recipients (Fig. 1).

The majority of individuals in the cohort were female (53.0%), older than 15 years (63.0%) and of non-white self-reported race/ethnicity (61.7%), had limited education (elementary/middle school 16.5%; elementary school 26.3%; illiterate/pre-school 29.5%), and were recipients of BFP (58.7%) (Table 1). At the baseline, most of the individuals lived in household with walls constructed of bricks/cement (81.6%), adequate sanitation (public network water 76.8% and public network sewage disposal 53.4%), public garbage collection (82.2%) and metered electricity supplies (86.2%). Most of the individuals lived in South/Southeast (52.2%) and Northeast regions (26.9%) (Table 1). The characteristics of non-recipients and recipients of the MCMVP were similar to the full study population. MCMVP recipients and non-recipients differ mainly in their household characteristics, such as water supply (SMD = 0.22) and garbage disposal (SMD = 0.29) (Table 1).

**Table 1:** Sociodemographic, household and geographic characteristics of the study population, 2010–2015

**Variables**			**MCMVP**				
	**Total (*N* = 24,584,768)**		**Non-recipients (*N* = 23,965,885)**		**Recipients (*N* = 618,883)**		
	***n***	**%**	***n***	**%**	***n***	**%**	**SMD**^**a**^
**Sociodemographic**							
Sex							0.08
Female	13,038,002	53.0	12,686,375	52.9	351,627	56.8	
Male	11,546,766	47.0	11,279,510	47.1	267,256	43.2	
Age							0.15
Up to 14 years	8,896,153	36.2	8,689,308	36.3	206,845	33.4	
15–29 years	5,523,583	22.5	5,363,691	22.4	159,892	25.8	
30–49 years	5,696,879	23.1	5,533,024	23.1	163,855	26.5	
50 or more years	4,268,967	17.4	4,185,720	17.4	83,247	13.5	
Missing	199,186	0.8	194,142	0.8	5,044	0.8	
Race/Ethnicity							0.01
White	8,437,346	34.3	8,228,913	34.3	208,433	33.7	
Non-white	15,161,147	61.7	14,775,996	61.7	385,151	62.2	
Missing	986,275	4.0	960,976	4.0	25,299	4.1	
Schooling							0.16
High school/College	6,044,746	24.6	5,858,451	24.4	186,295	30.1	
Elementary/Middle school (4—9 years)	4,063,877	16.5	3,950,759	16.5	113,118	18.3	
Elementary school (< 4 years)	6,462,260	26.3	6,316,233	26.4	146,027	23.6	
Illiterate/preschool	7,248,685	29.5	7,092,393	29.6	156,292	25.2	
Missing	765,200	3.1	748,049	3.1	17,151	2.8	
Benefit of the BFP							0.17
Non-recipient	10,154,358	41.3	9,846,189	41.1	308,169	49.8	
Recipient	14,430,410	58.7	14,119,696	58.9	310,714	50.2	
**Household**							
Walls construction material							0.04
Bricks/Cement	20,062,503	81.6	19,560,853	81.6	501,650	81.1	
Taipa/Wood/Other	3,553,994	14.5	3,475,585	14.5	48,409	12.7	
Missing	968,271	3.9	929,447	3.9	38,824	6.3	
Water supply							0.22
Public network	18,863,591	76.8	18,353,564	76.6	510,027	82.4	
Well/natural source/other	4,752,940	19.3	4,682,905	19.5	70,035	11.3	
Missing	968,237	3.9	929,416	3.9	38,821	6.3	
Waste disposal system							0.11
Public network	13,135,032	53.4	12,776,083	53.3	358,949	58.0	
Septic tank	8,466,343	34.4	8,280,046	34.5	186,297	30.1	
Ditch/other	1,098,795	4.5	1,074,368	4.5	24,427	4.0	
Missing	1,884,598	7.7	1,835,388	7.7	49,210	7.9	
Garbage disposal							0.29
Public collection system	20,211,477	82.2	19,665,113	82.0	546,364	88.3	
Burned/buried/other	3,404,995	13.9	3,371,300	14.1	33,695	5.4	
Missing	968,296	3.9	929,472	3.9	38,824	6.3	
Electricity supply							0.01
Electricity with counter	21,180,548	86.2	20,658,688	86.2	521,860	84.3	
Electricity without counter/other	2,435,971	9.9	2,377,772	9.9	58,199	9.4	
Missing	968,249	3.9	929,425	3.9	38,824	6.3	
**Geographical**							
Region							0.11
South/Southeast	12,842,319	52.2	12,542,295	52.3	300,024	48.5	
Northeast	6,601,899	26.9	6,435,978	26.8	165,921	26.8	
North	2,820,525	11.5	2,745,213	11.5	75,312	12.2	
Midwest	2,320,014	9.4	2,242,394	9.4	77,620	12.5	
Missing	11	0.0	5	0.0	6	0.0	

^a^Standardized Mean Difference

The NCDR was estimated to 11.83/100,000 pyr (95%CI = 11.58–12.08) for the total population (Table 2); and was higher among MCMVP recipients (13.32/100,000 pyr; 95%CI = 11.45–15.49) compared to non-recipients (11.72/100,000 pyr; 95%CI = 11.47–11.97), although the 95% CIs overlapped (Table 2); highest NCDRs were estimated among patients with multibacillary leprosy and grade 0 physical disabilities overall and within each of the MCMVP exposure groups (Table 2). Also, in sensitivity analysis for the 2006–2009 period, we observed higher NCDRs among those who subsequently became MCMV beneficiaries compared to non-beneficiaries (Table 2).

**Table 2:** Incidence of leprosy by clinical characteristics for the total population and by MCMVP receipt for the main analysis (2010–2015) and sensitivity analysis (2006–2009)

Clinical characteristics	*N*	%	Person-years at risk	Incidence per 100,000 person-years	95%CI
***Main Analysis***					
**Total population (*N* = 24,584,768)**	8,874		74,534,585.8	11.83	11.58–12.08
WHO operational classification					
Paucibacillary	3,020	34.0	74,534,588.3	4.04	3.90–4.19
Multibacillary	5,854	66.0	74,534,519.4	7.79	7.60–7.99
Physical disability at the diagnosis					
Grade 0	5,321	60.0	74,534,530.6	7.11	6.92–7.30
Grade 1	1,980	22.3	74,534,624.5	2.64	2.53–2.76
Grade 2	627	7.1	74,534,668.4	0.84	0.77–0.91
Missing	946	10.6			
**MCMVP**^**a**^					
**Non-recipients (*N* = 23,965,885)**	8,703		73,774,212.1	11.72	11.47–11.97
WHO operational classification					
Paucibacillary	2,963	34.1	73,774,235.4	4.08	3.94–4.23
Multibacillary	5,740	65.9	73,774,221.7	7.87	7.67–8.08
Physical disability at the diagnosis					
Grade 0	5,222	60.0	73,774,225.7	7.18	6.99–7.38
Grade 1	1,939	22.3	73,774,237.5	2.67	2.55–2.79
Grade 2	614	7.1	73,774,241.9	0.85	0.78–0.91
Missing	928	10.7			
**Recipients (*N* = 618,883)**	171		1,268,397.7	13.32	11.45–15.49
WHO operational classification					
Paucibacillary	57	33.3	1,268,397.7	4.49	3.47–5.83
Multibacillary	114	66.8	1,268,397.7	8.83	7.34–10.63
Physical disability at the diagnosis					
Grade 0	99	57.9	1,268,397.7	7.65	6.27–9.33
Grade 1	41	24.0	1,268,397.7	3.23	2.38–4.39
Grade 2	13	7.6	1,268,397.7	1.02	0.59–1.76
Missing	18	10.5			
***Sensitivity Analysis***^***b***^					
**Total population (*N* = 42,491,918)**	53,961		370,531,639	14.43	14.31–14.55
WHO operational classification					
Paucibacillary	21,794	40.4	370.532.031.1	5.85	5.77–5.93
Multibacillary	32,156	59.6	370.532.085.1	8.61	8.52–8.71
Missing	11	0.0			
Physical disability at the diagnosis					
Grade 0	33,640	62.3	370,531,986.1	9.03	8.93–9.12
Grade 1	11,170	20.7	370,532,310.1	3.00	2.95–3,06
Grade 2	3,174	5.9	370,532,441.1	0.85	0.82–0.88
Missing	5,977	11.1			
**MCMVP**^**a**^					
**Non-recipients (*N* = 41,767,722)**	53,063		365,873,869.1	14.37	14.25–14.50
WHO operational classification					
Paucibacillary	21,390	40.3	365,874,261.0	5.82	5.74–5.90
Multibacillary	31,662	59.7	365,874,315.0	8.59	8.49–8.68
Missing	11	0.0			
Physical disability at the diagnosis					
Grade 0	33,075	62.3	365,874,216.1	8.99	8.89–9.09
Grade 1	10,988	20.7	365,874,540.0	2.99	2.94–3.05
Grade 2	3,130	5.9	365,874,671.0	0.85	0.82–088
Missing	5.870	11.1			
**Recipients (*N* = 724,196)**	898		4,657,770.1	18.98	17.78–20.27
WHO operational classification					
Paucibacillary	404	45.0	4,657,770.1	8.54	7.74–9.43
Multibacillary	494	55.0	4,657,770.1	10.43	9.55–11.40
Physical disability at the diagnosis					
Grade 0	565	62.9	4,657,770.1	10.94	10.98–12.97
Grade 1	182	20.3	4,657,770.1	3.86	3.34–4.47
Grade 2	44	4.9	4,657,770.1	0.94	0.70–1.27
Missing	107	11.9			

^a^Minha Casa Minha Vida programme

^b^Sensitivity analysis considering the leprosy detection among those individuals who subsequently became MCMVP recipients before receiving the MCMVP benefit (i.e., from 2006 to 2009)

We estimated that, compared to those unexposed to MCMVP, individuals who received MCMVP benefits at 1.66-times the risk of become a leprosy case (95%CI = 1.34–2.06) (Table 3). In sensitivity analyses, the point estimated for the effect of MCMCP on leprosy reduced when we considered individuals actually moved to the new household after 6 months (HR = 1.53, 95%CI = 1.20–1.95) or 12 months (HR = 1.37, 95%CI = 1.05–1.78) after signing contract date. By stratifying by municipality size, we found that the relative risks comparing MCMVP recipients to non-recipients were elevated in municipalities with populations ≤ 300,000 inhabitants (HR = 1.95, 95%CI = 1.51–2.52) compared to those in municipalities with > 300,000 inhabitants (HR = 1.21, 95%CI = 0.82–1.78). Notably, the sensitivity analyses that included individuals with leprosy detected prior to receiving MCMVP benefits to non-beneficiaries was estimated to be a HR of 2.29 (95%CI = 1.93–2.72) (Table 3).

**Table 3:** Proportional Hazard Ratios of leprosy detection for total population and by subgroups, 2010–2015

				**MCMVP**^**a**^** receipt**	
	***N***	**%**	**Leprosy cases**	**HR**^**b**^	**95% CI**
**Main analysis**					
Total	18,936,721				
Non-recipients	18,484,587	97.6	6,058	1.00	
Recipients	452,134	2.4	119	1.66	1.34–2.06
**Sensitivity analysis**					
***Start of the exposure time***					
After 6 months of signing the contract	18,010,047				
Non-recipients	17,649,493	98.0	4,973	1.00	
Recipients	360,554	2.0	93	1.53	1.20–1.95
After 12 months of signing the contract	16,397,153				
Non-recipients	16,076,744	98.1	4,027	1.00	
Recipients	320,409	1.9	72	1.37	1.05–1.78
***Municipality’s population size***					
> 300,000 inhabitants	7,749,768				
Non-recipients	7,270,068	97.8	1,517	1.00	
Recipients	179,700	2.2	34	1.21	0.82–1.78
≤ 300,000 inhabitants	11,939,075				
Non-recipients	11,666,641	97.2	4,529	1.00	
Recipients	272,434	2.8	85	1.95	1.51–2.52
***Leprosy Risk prior MCMVP***					
Total	18,938,332				
Non-recipients	18,486,078	97.6	6,073	1.00	
Recipients	452,254	2.4	204	2.29	1.93–2.72

^a^Minha Casa Minha Vida programme

^b^Proportional Hazard Ratio (HR) estimated using Cox regression model weighted by IPTW

## Discussion

In this large study analysing data from over 24 million individuals followed for up to six years, we found a higher risk of leprosy for individuals who became MCMVP beneficiaries compared to their counterparts who did not receive the MCMVP housing. However, our results were not robust given also higher baseline leprosy risk among MCMVP beneficiaries.

Leprosy is strongly associated with social vulnerability [5, 8, 9], and prior evidence supports the positive impact of social protection policies in reducing leprosy burden, such as the cash transfer BFP [18, 19]. MCMVP was majorly implemented in urban centres with high population densities and precarious social environments in Brazil [20]. It is known that housing with better infrastructure, such as improved access to drinking water and adequate sanitation, can improve hygiene conditions [5, 21]. In addition, reduced household crowding may contribute to decreased contact between cohabitants and, consequently, reduced transmission risks [22, 23].

Given this context, we hypothesize that the increased risk of leprosy observed among the MCMVP beneficiaries in this study may, therefore, reflect reverse causality [24]. Further, our findings may suggest the programme is, in fact, reaching the most vulnerable individuals, as intended in its objectives. This is supported by our sensitivity analysis that demonstrated higher NCDRs among those individuals who subsequently became MCMVP recipients when compared to non-recipients at the same period (2006–2009). Supporting this hypothesis, the higher risk of leprosy (HR 2.29) among MCMVP beneficiaries even before receiving the benefit observed in another sensitivity analysis may reflect residual confounding factors related to structural poverty [8]. Although people enrolled in CadÚnico and, consequently, in our cohort, represent the poorest segment of the Brazilian population overall, among these, there are those considered extremely poor. Those are already more likely to be living under worse circumstances of deprivation [8], demanding a social assistance more effective.

It should also be noted that the short time span between the provision of housing and the detection of leprosy cases may not be sufficient for fully evaluating a causal impact between the exposure and the outcome. Although our study included the full six-year period with available data (2010–2015) and a median exposure time of 1.9 (interquartile range: 0.8 to 3.3) years for the MCMVP recipients, this length of exposure to new housing may not have been enough to overcome underlying social and health problems for people in poverty, especially extreme poverty, who were already susceptible to a higher incidence of leprosy. Sensitivity analysis also suggested reduced point estimates once we considered the follow-up of MCMVP to start after the 6 th of the 12 th month after signing the contract. In this way, we support the evidence that the MCMV programme was a social protection policy that contributed to mitigating the leprosy risk among beneficiaries over time.

Despite the large gaps that still exist in the epidemiological knowledge of leprosy, it is known that the main transmission for *M. leprae* occurs through droplets from a patient without treatment to a susceptible person, during a prolonged direct contact [4, 25]. However, the incubation period of the infection range from two to 20 years and signs or symptoms of the disease can take time to appear [4], therefore, the time of diagnosis is not actually the moment of the transmission/infection. Beyond that, since SINAN-leprosy registrations are made based on passive surveillance, one of our assumptions is that some leprosy patients notified after receiving the MCMVP could have become infected in the previous precarious housing environment and only became ill sometime later. Another assumption worth mentioning is that, although people have moved to new housing, their contact patterns within the homes may be similar, in certain circumstances, to their contact patterns in their old housing. For example, parent-to-child contacts are likely very similar regardless of the housing situation.

Certainly policymakers hope that social protection programmes that improve affordable housing for low-income families can reduce social inequalities and therefore benefit health outcomes. Regarding to the MCMVP, an assessment of the social ramifications of the programme from the creation of millions of new low-cost housing locations for poor families may be complex and limited. The findings of the literature related to economic evaluations of MCMV have shown that the programme still have gaps in some areas/municipalities, especially in the provision of quality housing, especially under different municipal administrations. It is important to evaluate housing policies strategies, since the people's ability to enjoy a healthy housing is directly and indirectly affect by offer and guarantee of policies of education, employability and health care [1].

### Strengths and limitations

This study provided a unique opportunity to study the effect of a social programme targeting housing on leprosy detection using The 100 Million Brazilian Cohort, a cohort covering the poorest half of the Brazilian population which represents a large and mighty resource of sociodemographic and health information. Furthermore, this is the first study assessing the effect of a social housing programme in leprosy detection. This analysis, primary and sensitivity results, supports the strength of the association between leprosy and social vulnerability and investigates other potential factors for reducing the leprosy burden. Nevertheless, this study presents limitations. Compulsory leprosy notification is mandatory in Brazil, but there is heterogeneity in the frequency and completeness of reporting [26]. Even with the improvement in the quality and completeness of SINAN information over time, underreported cases and lack of information still occur, mainly in the most vulnerable areas [26], which could influence on the number of leprosy cases among MCMVP recipients and, consequently, on the bias in our analysis. As The 100 Million Brazilian Cohort was built using data from national health and administrative registries that were not designed for this type of evaluation, the dataset did not have certain variables (e.g., health-seeking behaviour and proximity to health/social/urban services), a gap that could have contributed to residual confounding. Finally, the available data on the MCMVP does not guarantee that all family members in fact moved and/or remained in the new housing, which could also lead to information bias regarding the exposure to the programme and underestimating a potential positive impact of the programme.

## Conclusions

Our findings demonstrate that the MCMVP has yet to achieve an effect in reducing the burden of leprosy in the poorest population in Brazil. The higher risk of leprosy among MCMVP beneficiaries suggest the perpetuation of the cycles of poverty among these people, and that more social investments are needed to overcome this situation. We emphasize that social policies as the MCMVP should be improved and extended to the greatest possible number of deprived families. Social development has been central to leprosy control in developed countries [27] and, therefore, it is a key priority to reduce the incidence and burden of leprosy among low-income people. Future research should study the effect of the MCMVP on leprosy and explore whether the hypotheses explored in this work will be confirmed over a longer period of time.

## Data Availability

All data supporting this study were obtained from the Center for Data and Knowledge Integration for Health (Cidacs). These were licensed for exclusive use in the present study and, due to the privacy rules of the Brazilian Laws and Ethics Committee, are not openly available. Upon request with adequate justification and approval of an ethics committee, controlled access to data is considered and, if possible, allowed access. The data described in the manuscript, code book, and analytical code will be made available upon request to the corresponding author, E-mail: csst.camila@gmail.com. To access the data, each researcher should present a research project, ethical approval, and a data plan, to extract an unidentified/anonymized data set for analysis. Further information can be obtained at: https://cidacs.bahia.fiocruz.br/o-cidacs-e-a-lgpd/.

## Abbreviations

95% CI: 95% Confidence Interval
BFP: ‘*Bolsa Família*’ Programme
CadÚnico: *Cadastro Único para Programas Sociais*
CIDACS: Center for Data and Knowledge Integration for Health
FAR: *Fundo de Arrendamento Residencial*
HR: Hazard Ratios
IPTW: Inverse Probability of Receiving the Treatment
MCMVP: ‘*Minha Casa Minha Vida*’ Programme
MB: Multibacillary
NCDR: New Case Detection Rate
NIS: *Número de Identificação Social*
PB: Paucibacillary
PYR: Person-years at risk
PS: Propensity Scores
SINAN: *Sistema de Informação de Agravos de Notificação*
SMD: Standardized Mean Differences
